# Radioulnar Synostosis after Endomedullary Fixation of the Radius and Ulna in a Patient with Floating Elbow: A Case Report

**DOI:** 10.1055/s-0044-1779333

**Published:** 2024-05-15

**Authors:** Manuel Ricardo Medellin Rincon, Diana Camila Navarro Pimiento, Edwin Nicolas Ortegon Candela, Camila Muñoz Vanegas, Raul Ernesto Gonzalez Chavez

**Affiliations:** 1 Departamento de Cirurgia Ortopédica, Fundación Clinica Shaio, Bogotá, Colômbia

**Keywords:** craniocerebral trauma, forearm, forearm injuries, radial nerve, synostosis

## Abstract

Floating elbow is a complex and rare entity caused by high-energy trauma. In this paper, we present the case of a patient who suffered a traffic accident with severe head trauma, floating elbow (humeral diaphyseal fracture, radial proximal diaphyseal fracture, and ulnar segmental fracture) and radial nerve injury. Fixations were made with a humeral plate and intramedullary rods in the forearm. Although the outcome was satisfactory, radioulnar synostosis was identified in postoperative controls. Due to the neurological compromise, type of fractures, and stabilization selected, we believe that the use of forearm intramedullary rods for similar cases should be carefully evaluated.

## Introduction

Floating elbow is characterized by ipsilateral fractures of the humerus and one or both forearm bones. It has an incidence of 2–13% and commonly occurs in traffic accidents, 1 being associated with open fractures, neurovascular and soft-tissue injuries. 2 The prognosis is variable, depending upon the degree of injury and its management. 3 4 The treatment is initially focused on damage control (immobilization or temporary fixation), followed by definitive repairs.

There are currently several classifications which use anatomical or structural criteria for their definitions. 5 6 Due to the variability of the lesions, these classifications do not establish prognostic or treatment criteria for the decision-making process.

In this paper, we analyze a polytrauma patient, who was managed with radioulnar nailing and developed postoperative synostosis. After evaluating the clinical characteristics, we believe that intramedullary fixation should be used with caution, particularly if there are risk factors for synostosis (comminuted fractures, severe head trauma, delay in the final treatment).

## Clinical Case

**Fig. 1:**
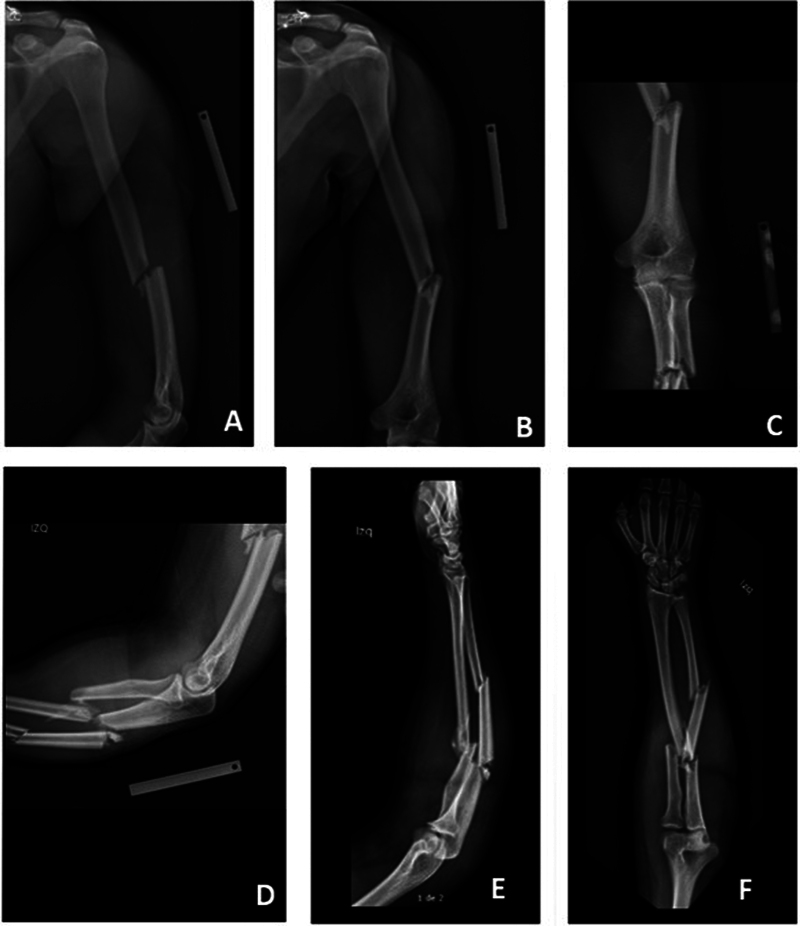
Initial radiological examination of the arm and forearm. The diaphyseal fracture of the humerus is observed in the lateral (A) and anteroposterior (B) planes. X-rays of the elbow showing the relationship between the humerus and forearm fractures in the anteroposterior (C) and lateral (D) planes. Segmental fracture of the ulna and fracture in the proximal third of the radius, in the lateral (E) and anteroposterior (F) planes.

A 19-years-old male patient was admitted to the emergency department presenting injuries after a traffic accident. He was driving a vehicle that collided at high speed against a cargo truck. At the initial assessment, severe head trauma (Glasgow 6/15) and multiple deformities in the left upper limb were documented. Brain tomography images revealed intracranial hypertension secondary to diffuse cerebral edema. Plain radiographs of the left upper limb displayed a shaft fracture of the humerus, segmental fracture of the ulna, and a short oblique shaft fracture of the radius ( Fig. 1 ).

Neurosurgical management was performed with decompressive craniotomy. The patient remained at intensive care unit 26 days due to the presence of diffuse axonal injury. The surgical stabilization of the fractures was postponed until general conditions improved, leaving a resolving motor aphasia as sequelae. After 27 days of the initial trauma, the definitive management of the fractures was performed.

**Fig. 2:**
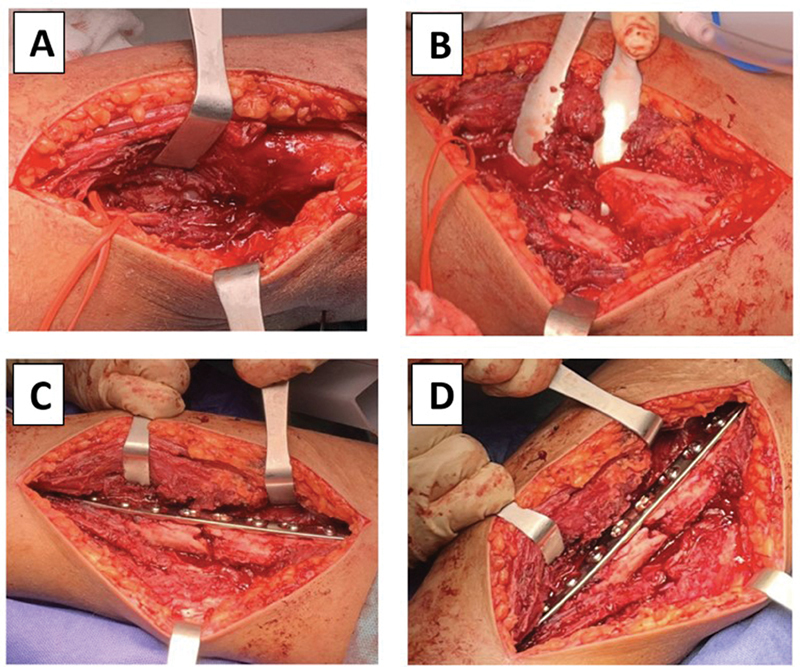
Intraoperative photographs. The radial nerve is observed continuous (A), presence of hypertrophic bone callus (B), stable reduction with 10-hole DCP plate (C, D).

**Fig. 3:**
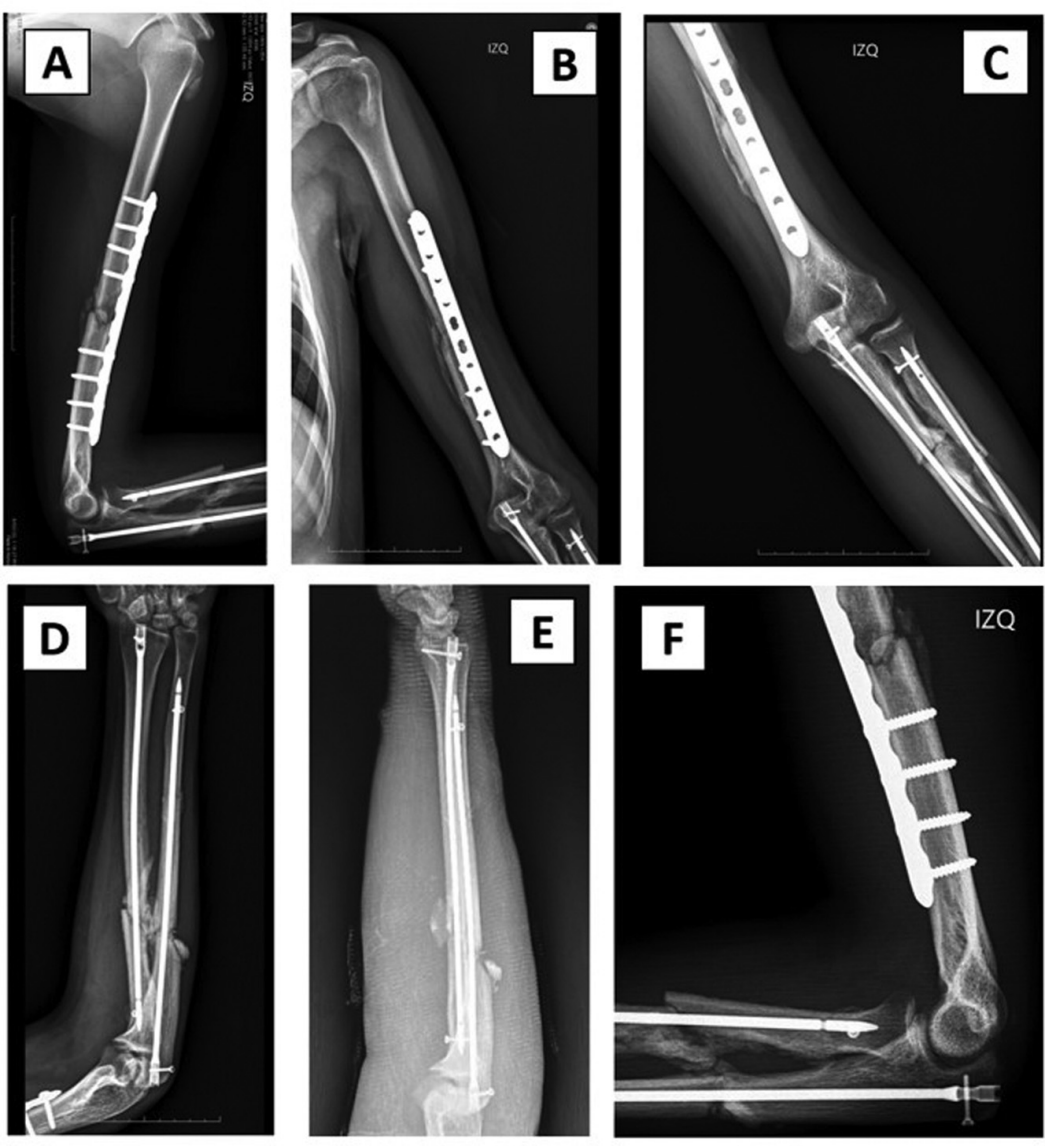
Postoperative radiographs. The fixation in the humerus with the LC-DCP plate is observed in the lateral (A) and anteroposterior (B) planes. X-rays of the elbow showing adequate restoration of the relationship between the humerus and forearm fractures in the anteroposterior (C) and lateral (F) planes. Stabilization of radius and ulna fractures with intramedullary nails in the anteroposterior (F) and lateral (E) planes.

During the surgical intervention, hypertrophic bone callus was documented and removed in the humerus. It was subsequently fixed with a narrow 4.5mm LC-DCP plate (Johnson & Johnson) ( Fig. 2 ). The radial nerve presented contusion without lesions. Fractures of the radius and ulna were stabilized with locked intramedullary nails (TREU-Instrumente GmbH). In the postoperative control the pronation/supination was 130°. The hand flexion was recovered, persisting wrist drop ( Fig. 3 ).

**Fig. 4:**
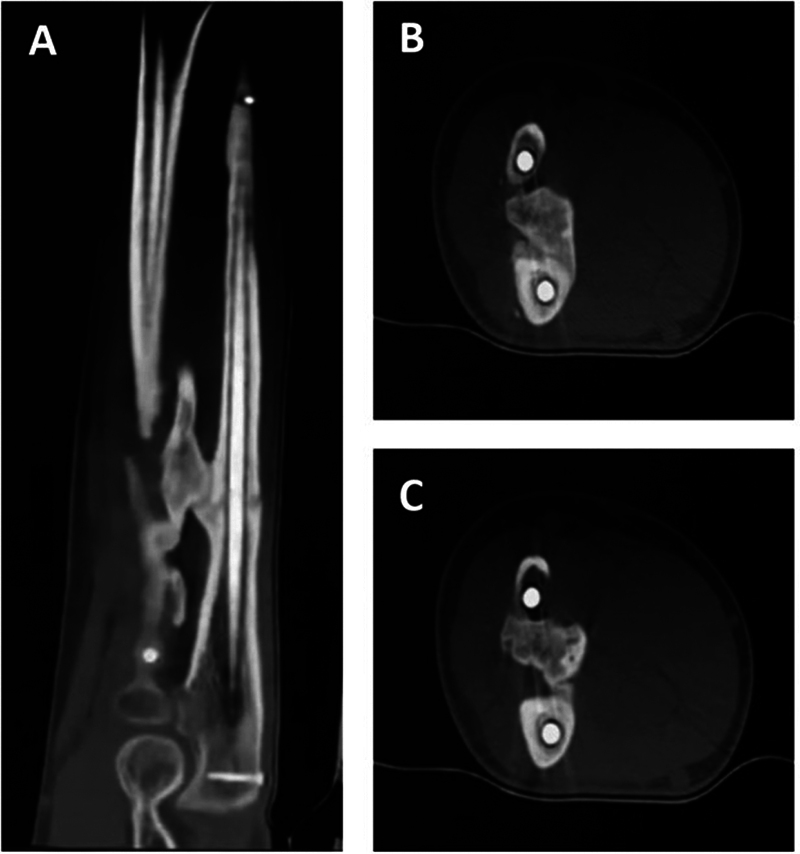
CT scan images displaying the radioulnar synostosis at the junction of the middle third with the proximal third of the diaphysis. Sagittal slices (A), axial slices (B, C).

**Fig. 5:**
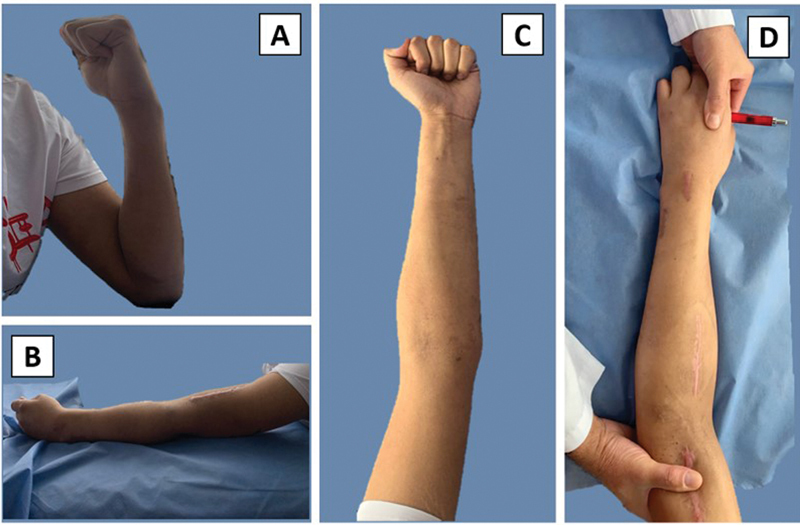
Physical examination at last follow up. A range of motion of the elbow can be seen with adequate flexion (A) and extension (B). Maximum supination within the tolerable range (C), pronation with blockage, presenting humeral compensation (D).

At 3 months follow-up a decrease in pronation/supination to 58° was documented, associated to the presence of radioulnar synostosis in diagnostic images ( Fig. 4 ). At last follow-up (8 months after osteosynthesis), progressive recovery of the radial nerve lesion has been observed. After applying the quickDASH questionnaire, a disability score of 30% was obtained and therefore, considering the clinical course, no additional surgical management has been indicated ( Fig. 5 ).

This case report was approved by the ethics committee under number CEI-115 - Acta 359 on April 28, 2023, and the patient signed the informed consent form.

## Discussion

Reports of floating elbow are not frequent in the literature and there is no consensus regarding treatment. Many papers describe different surgical approaches based on simple fracture lines and therefore, they are not applicable in all scenarios. 6

In the current case a deferred management of the lesions was necessary due to the patient's condition. Resection of hypertrophic callus was performed in the humerus, followed by exploration of the radial nerve and plate fixation. For the treatment of forearm fractures, closed management with intramedullary nailing was selected, although this is not frequently described.

In most reports, the use of locking plates is preferred to manage the forearm fractures. 5 6 In a meta-analysis performed by Ditsios et al., 7 258 floating elbow cases were identified. In those cases, the forearm fractures were mainly stabilized with plates (70.9%), followed by external fixation and immobilization (8.9% and 8.5% respectively), while intramedullary nails were used in only 4.7% of cases.

Studies that have compared the use of intramedullary nails vs plates in forearm fractures have not found higher rates of radioulnar synostosis. 8 However, the comparison is often made with simple fractures, which could explain these results. The use of intramedullary nails is not frequently described in comminuted fractures, and the studies that mention their use for floating elbows do not clearly report the treatment outcome.

A review by Bergeron et al. 9 showed that forearm fractures may develop radioulnar synostosis in 1.2% to 6% of the cases. According to some reports, this percentage can increase to 18% in patients with traumatic brain injury, 30% in patients with polytrauma, and up to 39% when the treatment has to be delayed.

The fracture patterns may also contribute to synostosis. In the patient here presented, factors such as comminution, fractures at the same level, and the position of the fragments make correct reduction more difficult, increasing the risk for non-union or improper-union.

Although it is not clear if intramedullary nailing is an isolated factor for radioulnar fusion in forearm fractures, we believe that in cases like the one presented, the combination of clinical characteristics (traumatic brain injury, late management, comminuted fractures), and the fixation with nails may increase the risks to develop synostosis.

Considering the relative stability of intramedullary nails and the risk of synostosis, their use for the treatment of radius and ulna fractures should be carefully evaluated. Several conditions such as polytrauma may also increase the risks and therefore, we believe that these devices should be preferred in the treatment of simple fracture patterns.
