# Supplementary material for: Radioulnar Synostosis after Endomedullary Fixation of the Radius and Ulna in a Patient with Floating Elbow: A Case Report
Source: Rev Bras Ortop (Sao Paulo). 2024 May 15;59(Suppl 2):e207–11. doi: 10.1055/s-0044-1779333 (PMC11679606; doi:10.1055/s-0044-1779333)
Supplement: Related texts

# Research article: Sinostose radioulnar após fixação endomedular do rádio e ulna em paciente com cotovelo flutuante: Relato de caso

**Keywords:** antebraço, nervo radial, sinostose, traumatismos craniocerebrais, traumatismos do antebraço

## Abstract

O cotovelo flutuante é um quadro complexo e raro, causado por trauma de alta energia. Neste artigo, apresentamos o caso de um paciente que sofreu um acidente de trânsito com trauma craniano grave, cotovelo flutuante (fratura diafisária do úmero, fratura diafisária proximal do rádio e fratura segmentar da ulna) e lesão no nervo radial. Foram realizadas fixações com uma placa umeral e hastes intramedulares no antebraço. Embora o resultado tenha sido satisfatório, a sinostose radioulnar foi identificada nos controles pós-operatórios. Devido ao comprometimento neurológico, ao tipo de fraturas e à estabilização selecionada, acreditamos que o uso de hastes intramedulares no antebraço para casos semelhantes deve ser cuidadosamente avaliado.

## Introdução

O cotovelo flutuante é caracterizado por fraturas ipsilaterais do úmero e de um ou ambos os ossos do antebraço. Tem incidência de 2 a 13% e ocorre comumente em acidentes de trânsito, 1 estando associado a fraturas expostas, lesões neurovasculares e de tecidos moles. 2 O prognóstico é variável, dependendo do grau da lesão e do seu manejo. 3 4 O tratamento é inicialmente focado no controle dos danos (imobilização ou fixação temporária), seguido de reparos definitivos.

Atualmente existem diversas classificações que utilizam critérios anatômicos ou estruturais para suas definições. 5 6 Devido à variabilidade das lesões, essas classificações não estabelecem critérios prognósticos ou de tratamento para a tomada de decisão.

Neste trabalho analisamos um paciente politraumatizado, tratado com haste radioulnar e que desenvolveu sinostose pós-operatória. Após avaliação das características clínicas, acreditamos que a fixação intramedular deve ser utilizada com cautela, principalmente se houver fatores de risco para sinostose (fraturas cominutivas, traumatismo cranioencefálico grave, atraso no tratamento final).

## Caso Clínico

Paciente do sexo masculino, 19 anos, deu entrada no pronto-socorro apresentando lesões após acidente de trânsito. Ele dirigia um veículo que colidiu em alta velocidade contra um caminhão de carga. Na avaliação inicial foram documentados traumatismo cranioencefálico grave (Glasgow 6/15) e múltiplas deformidades em membro superior esquerdo. As imagens de tomografia cerebral revelaram hipertensão intracraniana secundária a edema cerebral difuso. Radiografias simples do membro superior esquerdo mostraram uma fratura diafisária do úmero, fratura segmentar da ulna e uma fratura curta oblíqua da diáfise do rádio ( Fig. 1 ).

**Fig. 1:** Exame radiológico inicial do braço e antebraço. A fratura diafisária do úmero é observada nos planos lateral (A) e ântero-posterior (B). Radiografias do cotovelo mostrando a relação entre as fraturas do úmero e do antebraço nos planos ântero-posterior (C) e lateral (D). Fratura segmentar da ulna e fratura do terço proximal do rádio, nos planos lateral (E) e ântero-posterior (F).

O manejo neurocirúrgico foi realizado com craniotomia descompressiva. O paciente permaneceu 26 dias na unidade de terapia intensiva devido à presença de lesão axonal difusa. A estabilização cirúrgica das fraturas foi adiada até a melhora do quadro geral, deixando como sequela a remissão da afasia motora. Após 27 dias do trauma inicial, foi realizado o manejo definitivo das fraturas.

Durante a intervenção cirúrgica foi documentado e removido calo ósseo hipertrófico no úmero. Posteriormente foi fixado com uma placa LC-DCP estreita de 4,5 mm (Johnson & Johnson) ( Fig. 2 ). O nervo radial apresentava contusão sem lesões. As fraturas do rádio e da ulna foram estabilizadas com hastes intramedulares bloqueadas (TREU-Instrumente GmbH). No controle pós-operatório a pronação/supinação foi de 130°. A flexão da mão foi recuperada, persistindo a queda do punho ( Fig. 3 ).

**Fig. 2:** Fotografias intraoperatórias. Observa-se nervo radial contínuo (A), presença de calo ósseo hipertrófico (B), redução estável com placa DCP de 10 furos (C, D).

**Fig. 3:** Radiografias pós-operatórias. A fixação no úmero com a placa LC-DCP é observada nos planos lateral (A) e ântero-posterior (B). Radiografias do cotovelo mostrando restauração adequada da relação entre fraturas do úmero e do antebraço nos planos ântero-posterior (C) e lateral (F). Estabilização de fraturas de rádio e ulna com hastes intramedulares nos planos anteroposterior (F) e lateral (E).

Aos 3 meses de seguimento foi documentada uma diminuição da pronação/supinação para 58°, associada à presença de sinostose radioulnar nas imagens diagnósticas ( Fig. 4 ). No último seguimento (8 meses após a osteossíntese), foi observada recuperação progressiva da lesão do nervo radial. Após aplicação do questionário quickDASH obteve-se escore de incapacidade de 30% e portanto, considerando a evolução clínica, não foi indicado tratamento cirúrgico adicional ( Fig. 5 ).

**Fig. 4:** Imagens de tomografia computadorizada mostrando sinostose radioulnar na junção do terço médio com o terço proximal da diáfise. Cortes sagitais (A), cortes axiais (B, C).

**Fig. 5:** Exame físico no último acompanhamento. Uma amplitude de movimento do cotovelo pode ser vista com flexão (A) e extensão (B) adequadas. Supinação máxima dentro da faixa tolerável (C), pronação com bloqueio, apresentando compensação umeral (D).

Este relato de caso foi aprovado pelo comitê de ética sob número CEI-115 - Acta 359 em 28 de abril de 2023, e o paciente assinou o Termo de Consentimento Livre e Esclarecido.

## Discussão

Relatos de cotovelo flutuante não são frequentes na literatura e não há consenso quanto ao tratamento. Muitos artigos descrevem diferentes abordagens cirúrgicas baseadas em linhas de fratura simples e, portanto, não são aplicáveis em todos os cenários. 6

No presente caso foi necessário um manejo diferido das lesões devido ao estado do paciente. Foi realizada ressecção do calo hipertrófico no úmero, seguida de exploração do nervo radial e fixação da placa. Para o tratamento das fraturas do antebraço foi selecionado o manejo fechado com haste intramedular, embora isso não seja frequentemente descrito.

Na maioria dos relatos, o uso de placas bloqueadas é preferido para o manejo das fraturas do antebraço. 5 6 Em metanálise realizada por Ditsios et al., 7 foram identificados 258 casos de cotovelo flutuante. Nesses casos, as fraturas do antebraço foram estabilizadas principalmente com placas (70,9%), seguidas de fixação externa e imobilização (8,9% e 8,5% respectivamente), enquanto hastes intramedulares foram utilizadas em apenas 4,7% dos casos.

Estudos que compararam o uso de hastes intramedulares versus placas em fraturas do antebraço não encontraram taxas maiores de sinostose radioulnar. 8 Porém, muitas vezes a comparação é feita com fraturas simples, o que poderia explicar esses resultados. O uso de hastes intramedulares não é descrito com frequência nas fraturas cominutivas e os estudos que mencionam seu uso em cotovelos flutuantes não relatam com clareza o resultado do tratamento.

Uma revisão por Bergeron et al. 9 mostrou que as fraturas do antebraço podem desenvolver sinostose radioulnar em 1,2% a 6% dos casos. Segundo alguns relatos, esse percentual pode aumentar para 18% em pacientes com traumatismo cranioencefálico, 30% em pacientes com politraumatismo e até 39% quando o tratamento precisa ser adiado.

Os padrões de fratura também podem contribuir para a sinostose. No paciente aqui apresentado, fatores como cominuição, fraturas no mesmo nível e posição dos fragmentos dificultam a redução correta, aumentando o risco de não consolidação ou consolidação inadequada.

Embora não esteja claro se a haste intramedular é um fator isolado para fusão radioulnar nas fraturas do antebraço, acreditamos que em casos como o apresentado, a combinação de características clínicas (traumatismo cranioencefálico, manejo tardio, fraturas cominutivas) e a fixação com as unhas podem aumentar os riscos de desenvolver sinostose.

Considerando a relativa estabilidade das hastes intramedulares e o risco de sinostose, seu uso no tratamento de fraturas de rádio e ulna deve ser cuidadosamente avaliado. Diversas condições, como o politraumatismo, também podem aumentar os riscos e, portanto, acreditamos que estes dispositivos devem ser preferidos no tratamento de padrões de fratura simples.
